# Supplementary material for: Multiple distinct metastatic cell states are induced by epithelial-mesenchymal plasticity
Source: bioRxiv. 2026 Jul 24:2025.08.15.670583. Preprint. [Version 2] doi: 10.1101/2025.08.15.670583 (PMC13419502; doi:10.1101/2025.08.15.670583)
Supplement: Supplementary file 1 [file NIHPP2025.08.15.670583v2-supplement-1.pdf]

bioRxiv preprint doi: https://doi.org/10.1101/2025.08.15.670583; this version posted July 24, 2026. The copyright holder for this preprint (which was not certified by peer review) is the author/funder, who has granted bioRxiv a license to display the preprint in perpetuity. It is made available under a CC-BY 4.0 International license.

Figure S1

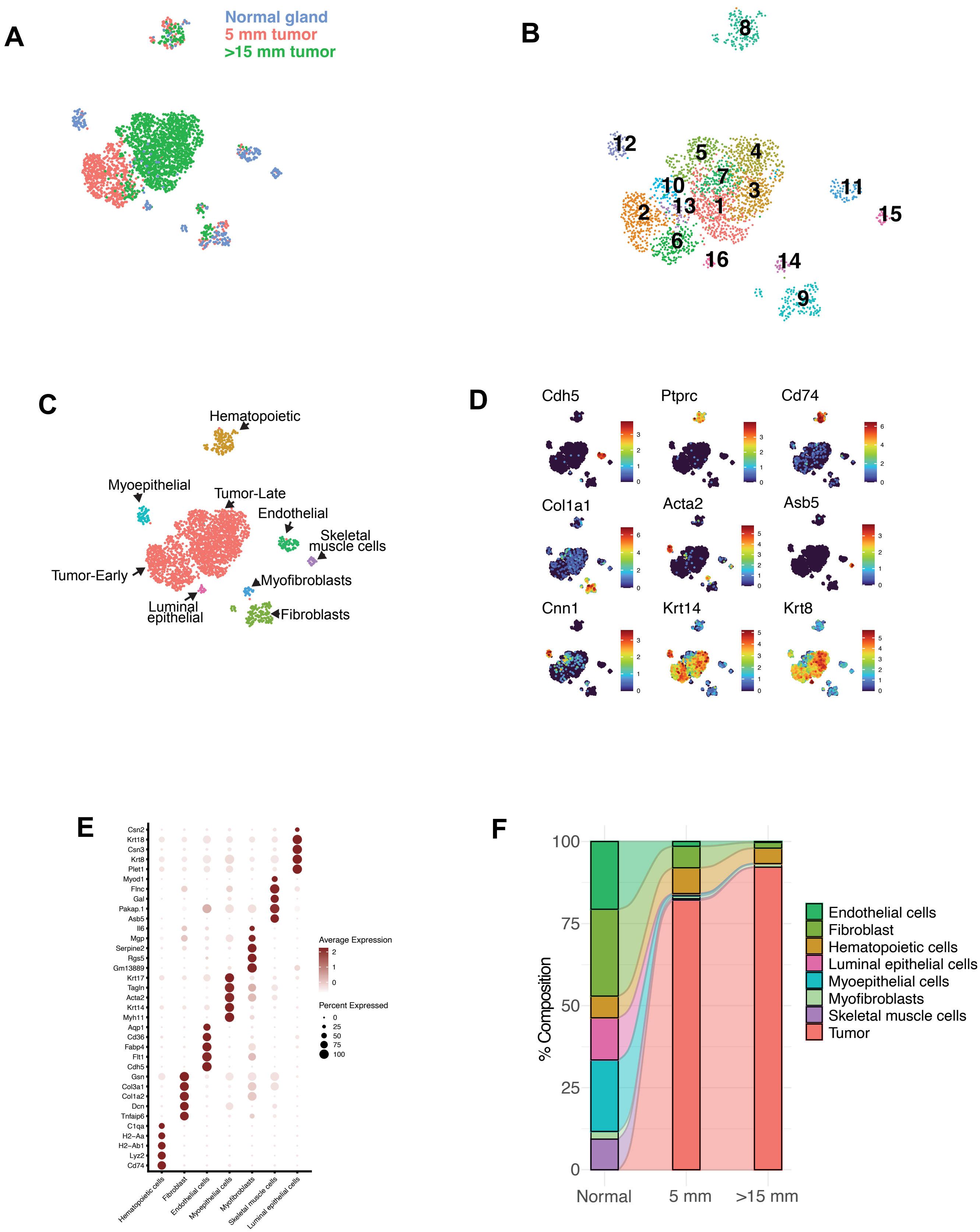

bioRxiv preprint doi: https://doi.org/10.1101/2025.08.15.670583; this version posted July 24, 2026. The copyright holder for this preprint (which was not certified by peer review) is the author/funder, who has granted bioRxiv a license to display the preprint in perpetuity. It is made available under a CC-BY 4.0 International license.

Figure S1, continued

G

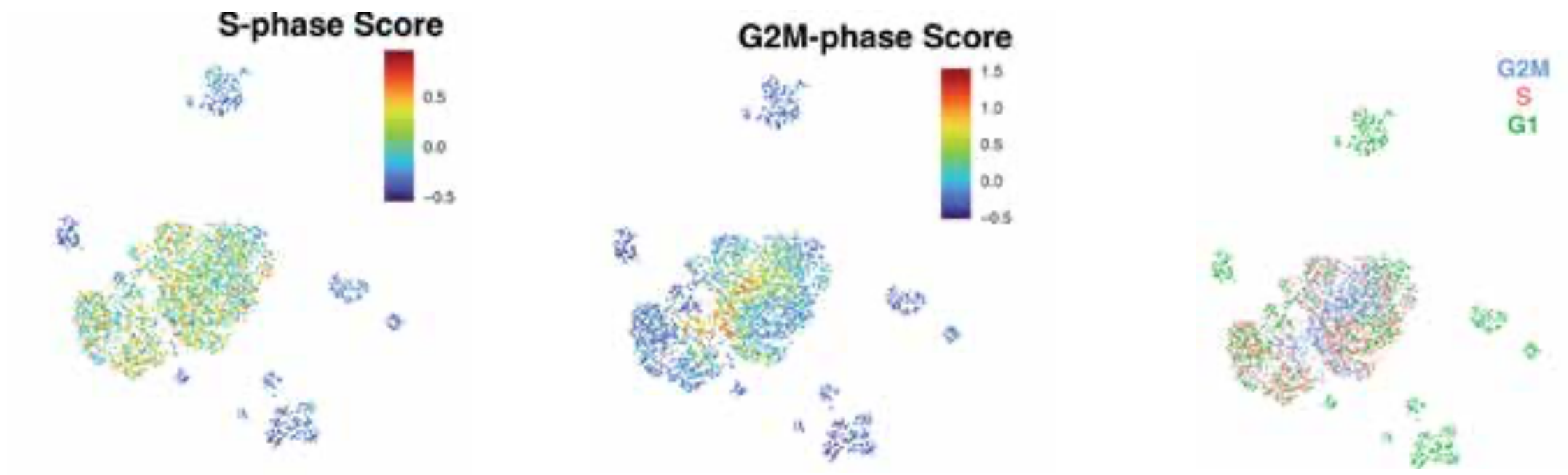

H

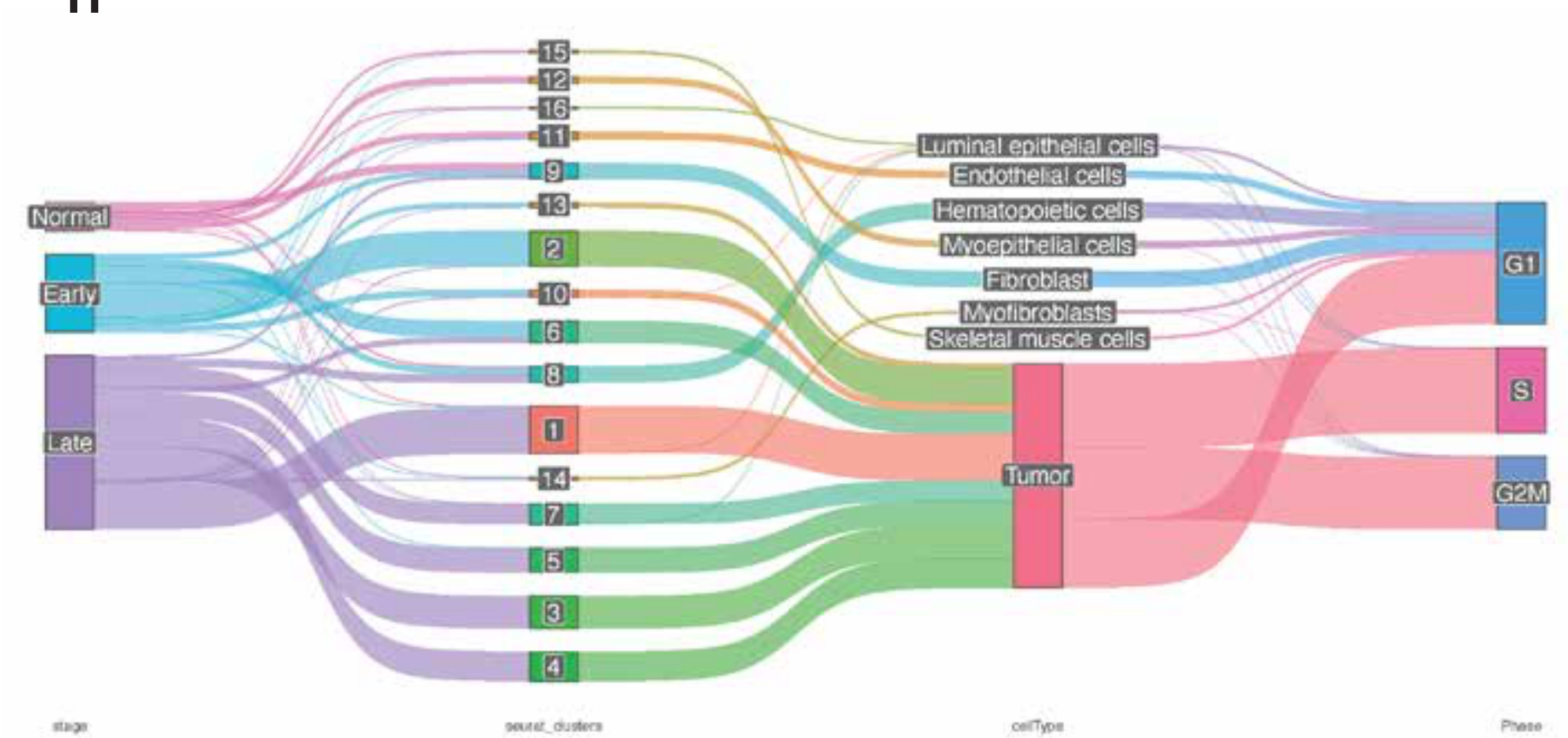

bioRxiv preprint doi: https://doi.org/10.1101/2025.08.15.670583; this version posted July 24, 2026. The copyright holder for this preprint (which was not certified by peer review) is the author/funder, who has granted bioRxiv a license to display the preprint in perpetuity. It is made available under a CC-BY 4.0 International license.

Figure S1, continued

I

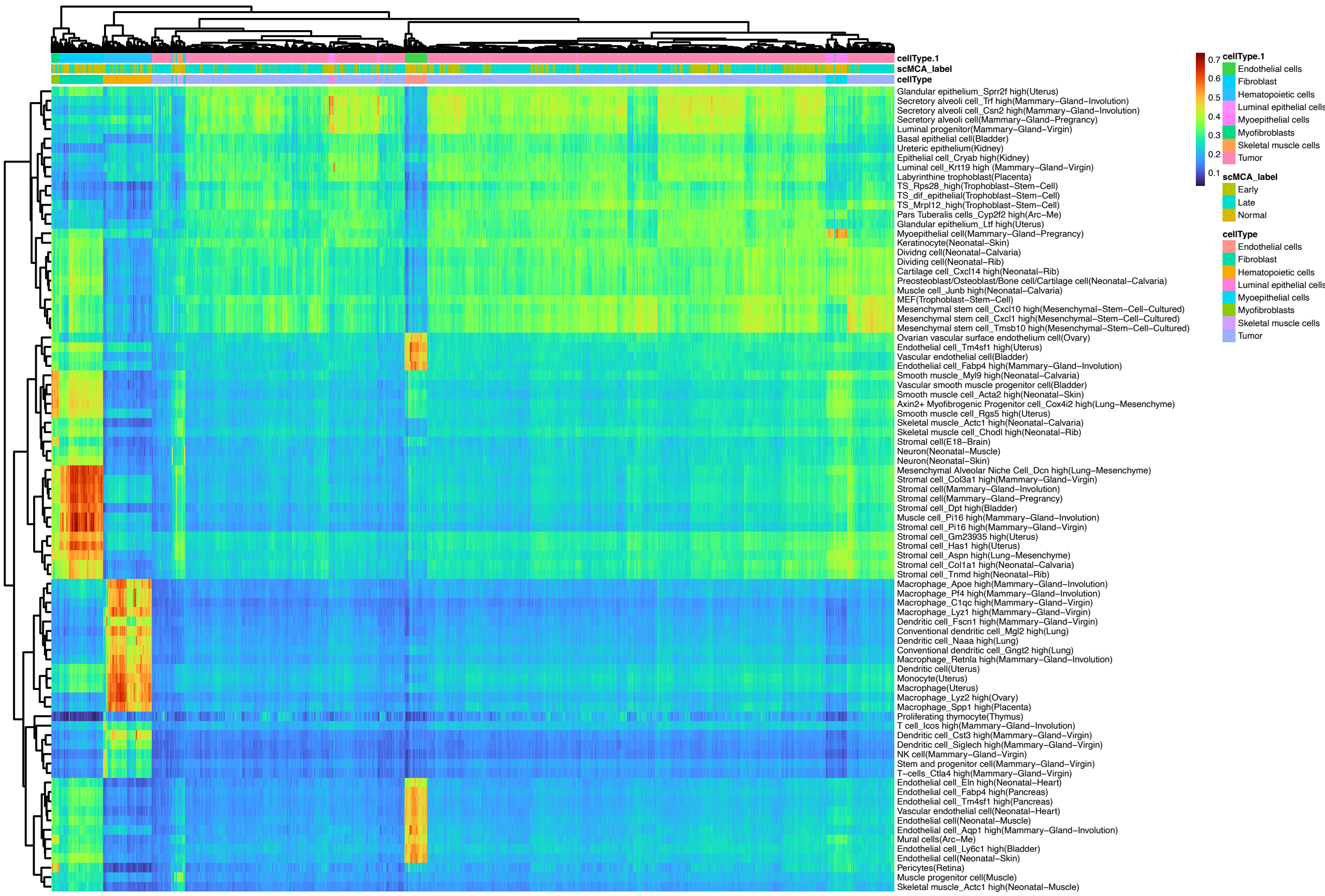

bioRxiv preprint doi: https://doi.org/10.1101/2025.08.15.670583; this version posted July 24, 2026. The copyright holder for this preprint (which was not certified by peer review) is the author/funder, who has granted bioRxiv a license to display the preprint in perpetuity. It is made available under a CC-BY 4.0 International license.

Figure S1, continued

J

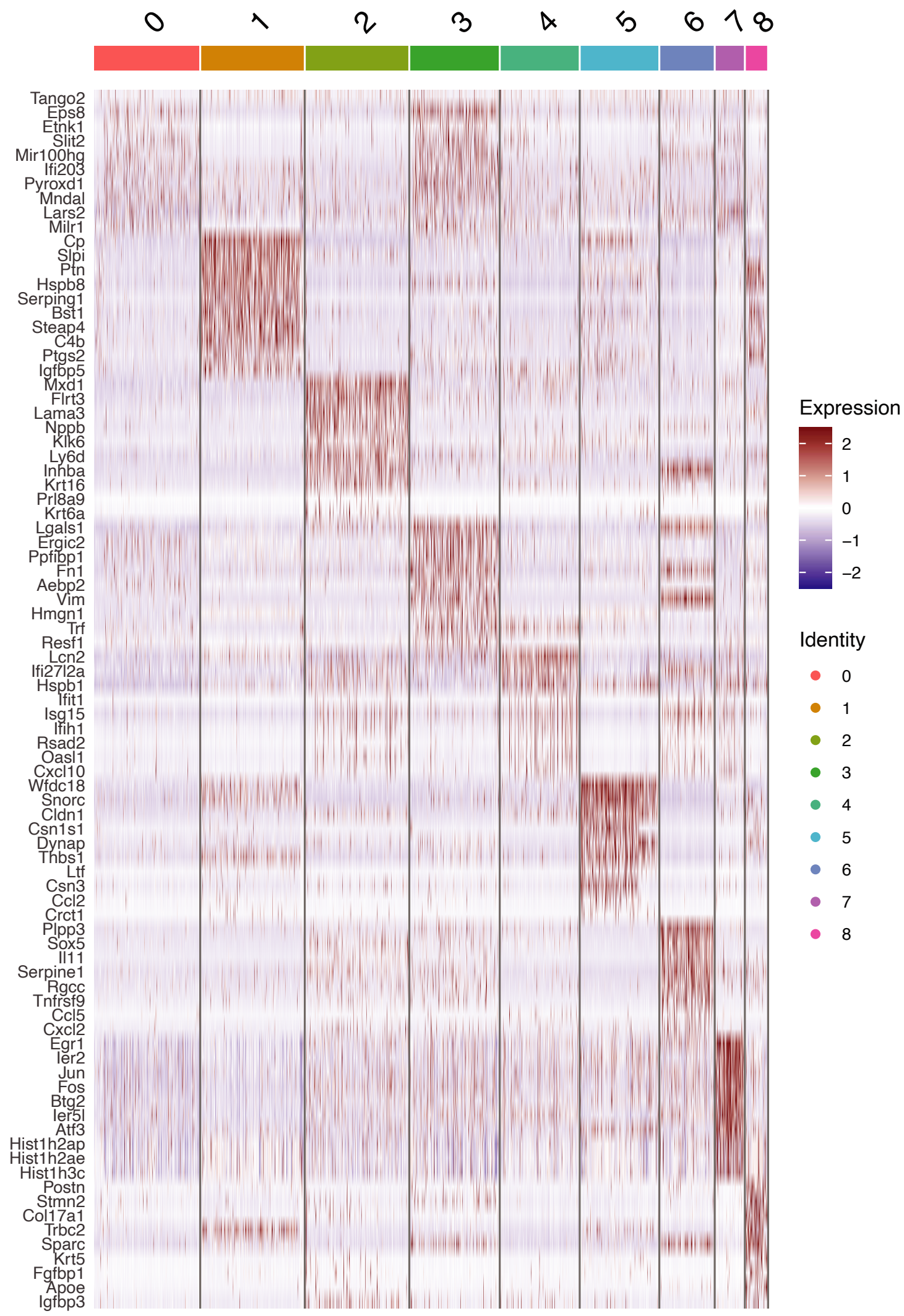

K

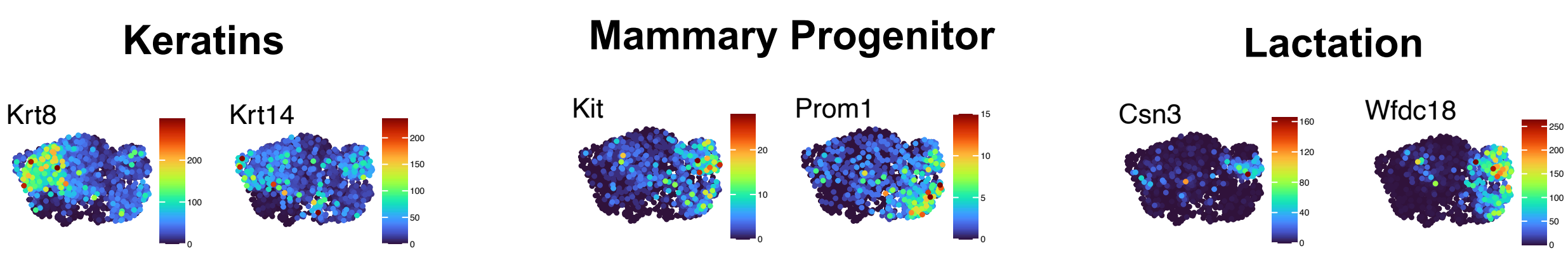

L

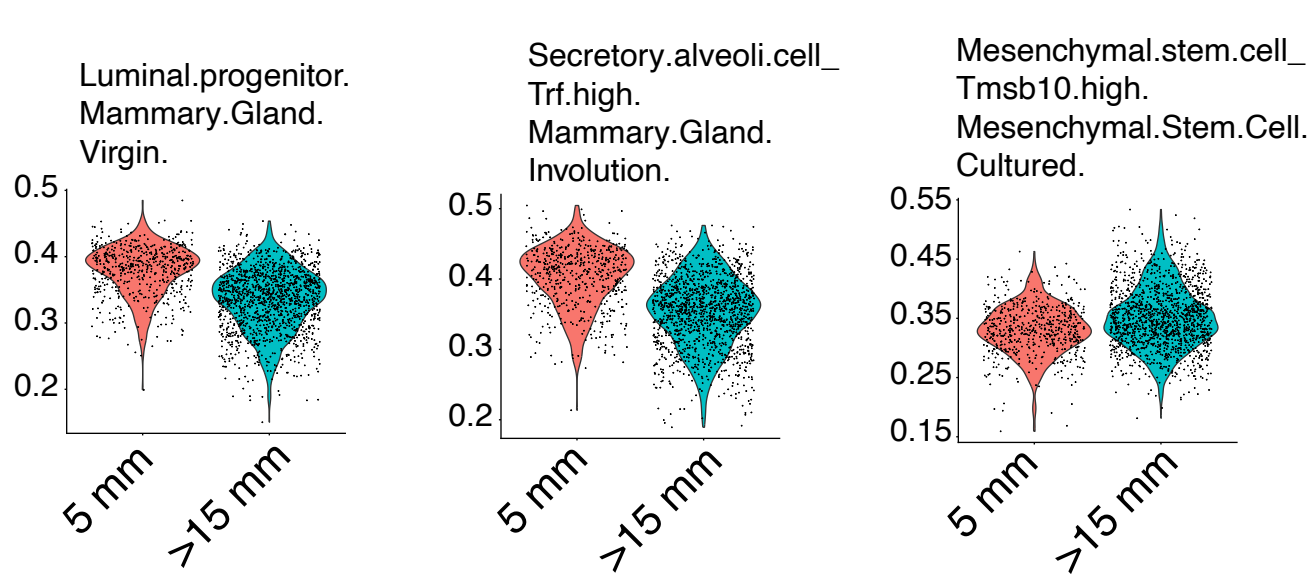

bioRxiv preprint doi: https://doi.org/10.1101/2025.08.15.670583; this version posted July 24, 2026. The copyright holder for this preprint (which was not certified by peer review) is the author/funder, who has granted bioRxiv a license to display the preprint in perpetuity. It is made available under a CC-BY 4.0 International license.

Figure S1. Related to C3-TAg tumor progression generates extensive EMT-associated transcriptional heterogeneity.

A. UMAP showing cells from normal mammary gland, 5 mm and >15 mm tumors annotated by sample source.

B. UMAP showing cell clusters from normal mammary gland, 5 mm and >15 mm tumors annotated by Seurat cluster.

C. UMAP showing cells from normal mammary gland, 5 mm and >15 mm tumors annotated by cell type.

D. UMAP showing the expression of cell lineage specific genes in cells from normal mammary gland, 5 mm and >15 mm tumors annotated by cell type.

E. Bubble plot showing the expression of marker genes in the indicated normal cell lineages.

F. Alluvial stacked bar plot showing the percentage of indicated cell types in normal mammary gland, 5 mm and > 15 mm tumors.

G. UMAPs showing the cell cycle phase of cells from normal mammary gland, 5 mm and >15 mm tumors annotated by sample source.

H. Alluvial plot showing the distribution of cells from normal mammary gland, 5 mm and >15 mm tumors.

I. Heatmap showing the similarity of the indicated cells to normal cell types from a mouse scRNA-seq atlas.

J. Heatmap showing the expression of top genes differentially expressed between clusters.

K. UMAPs showing the expression of representative luminal progenitor, lactation-associated and basal and genes in 5 mm and >15 mm tumor cells.

L. Quantification compates the similarity of 5 mm and >15 mm tumor cells to luminal progenitor, secretory alveolar and mesenchymal stem cells.

bioRxiv preprint doi: https://doi.org/10.1101/2025.08.15.670583; this version posted July 24, 2026. The copyright holder for this preprint (which was not certified by peer review) is the author/funder, who has granted bioRxiv a license to display the preprint in perpetuity. It is made available under a CC-BY 4.0 International license.

Figure S2

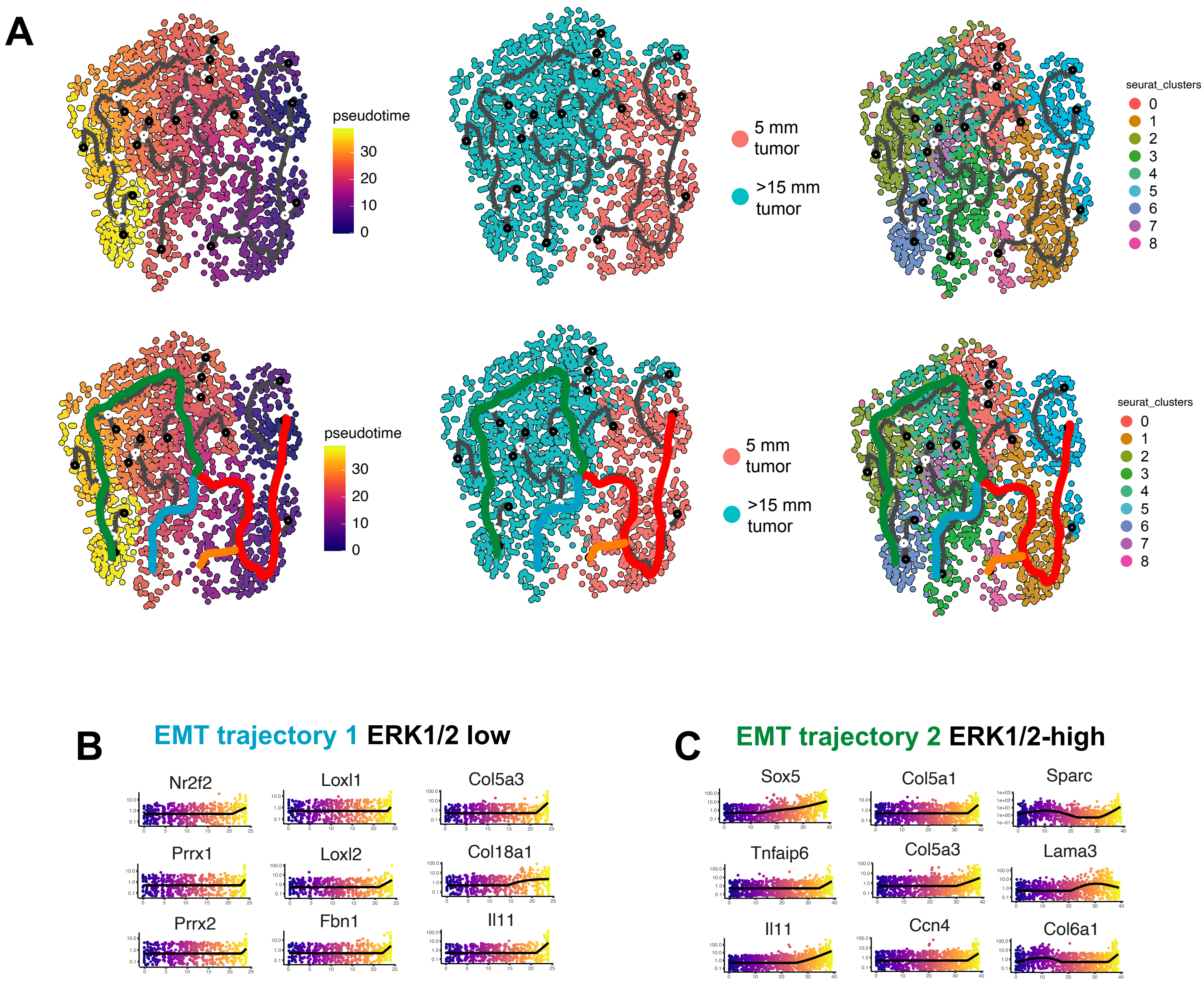

bioRxiv preprint doi: https://doi.org/10.1101/2025.08.15.670583; this version posted July 24, 2026. The copyright holder for this preprint (which was not certified by peer review) is the author/funder, who has granted bioRxiv a license to display the preprint in perpetuity. It is made available under a CC-BY 4.0 International license.

Figure S2, continued

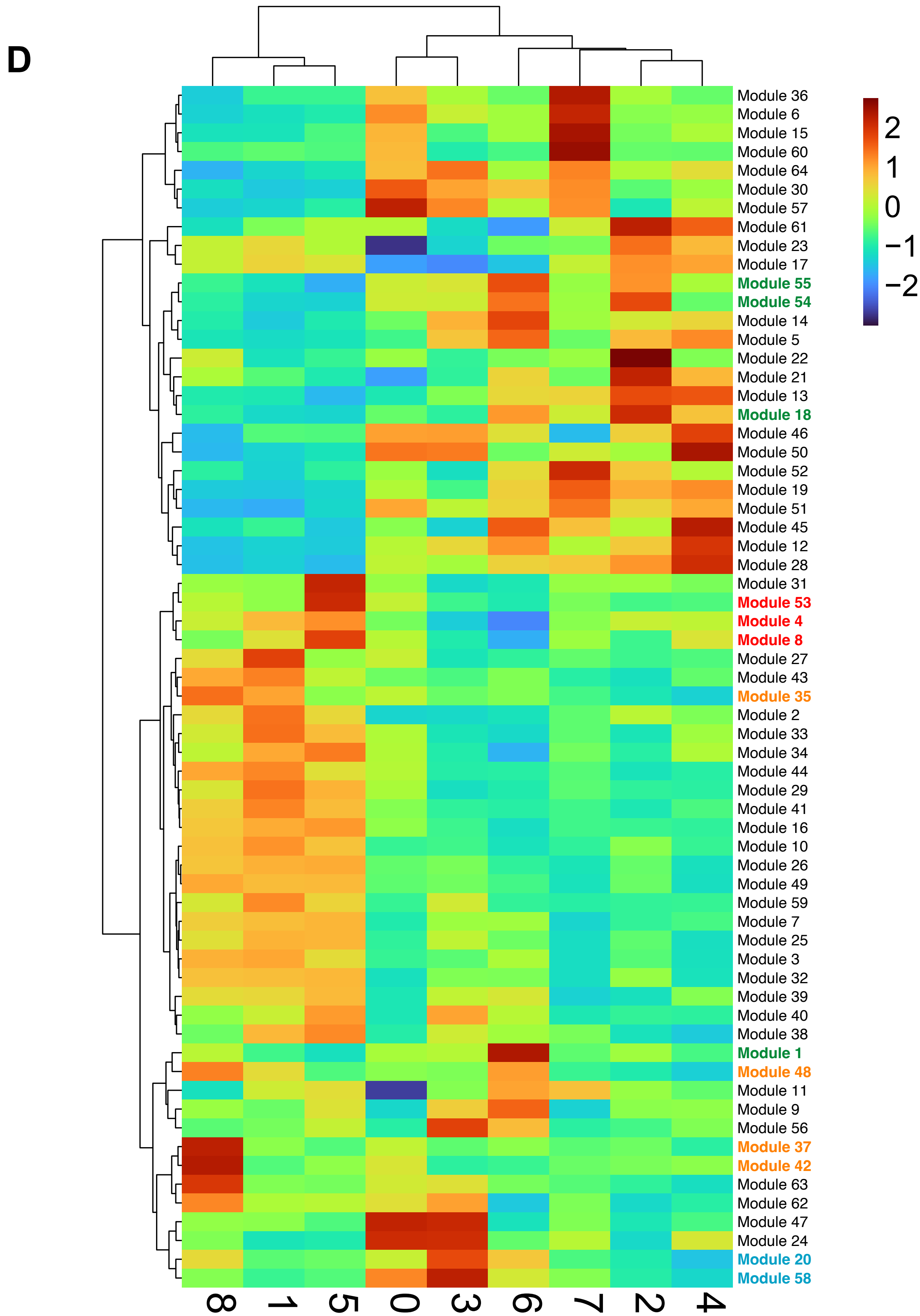

bioRxiv preprint doi: https://doi.org/10.1101/2025.08.15.670583; this version posted July 24, 2026. The copyright holder for this preprint (which was not certified by peer review) is the author/funder, who has granted bioRxiv a license to display the preprint in perpetuity. It is made available under a CC-BY 4.0 International license.

Figure S2, continued

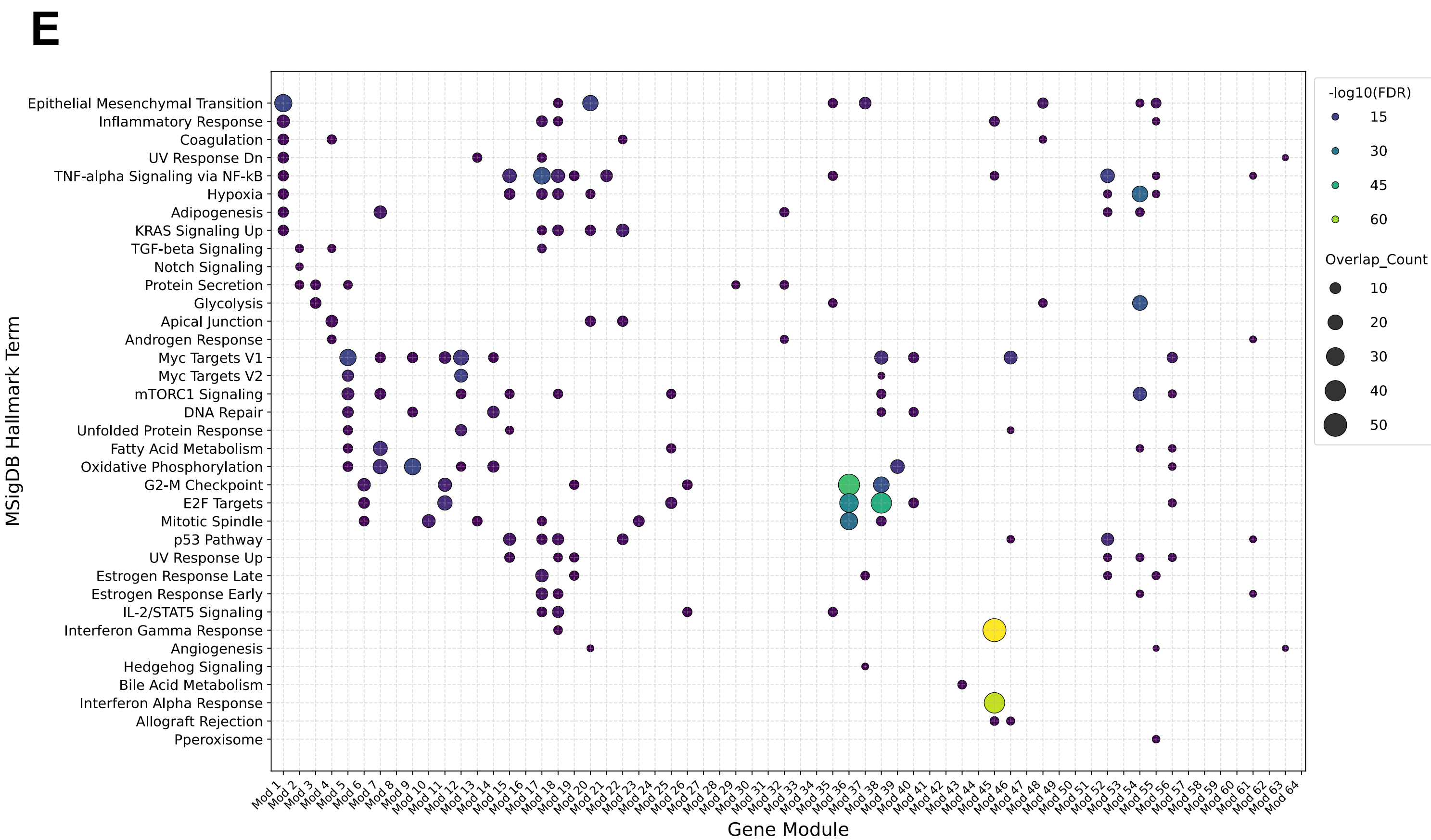

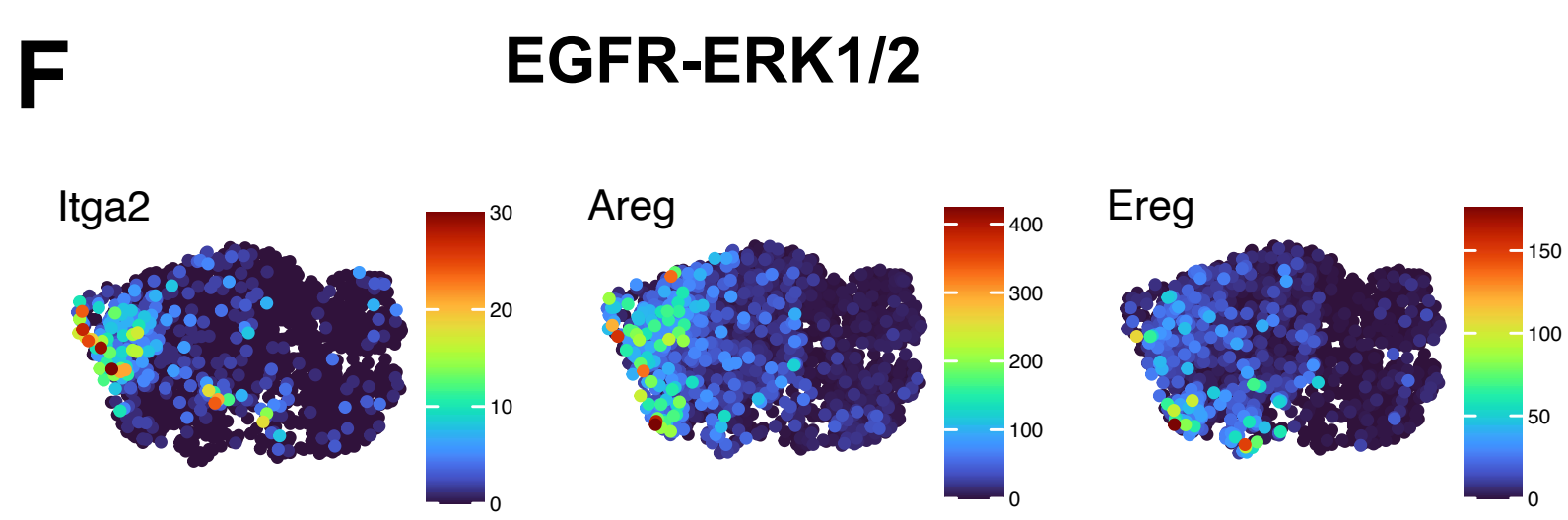

bioRxiv preprint doi: https://doi.org/10.1101/2025.08.15.670583; this version posted July 24, 2026. The copyright holder for this preprint (which was not certified by peer review) is the author/funder, who has granted bioRxiv a license to display the preprint in perpetuity. It is made available under a CC-BY 4.0 International license.

Figure S2. Related to EMT-associated tumor cell heterogeneity emerges along two transcriptional trajectories during C3-TAg tumor progression.

A. Monocle3 trajectory analysis of tumor cells from 5-mm and >15-mm C3-TAg tumors shown in Fig. 2A-C. Top row shows the raw trajectory analysis. Bottom row shows the overlay of the trunk and branching into EMT trajectory 1 and EMT trajectory 2.

B. Expression of representative mammary lineage, epithelial, and mesenchymal-associated genes across pseudotime for EMT trajectory 1.

C. Expression of representative mammary lineage, epithelial, and mesenchymal-associated genes across pseudotime for EMT trajectory 2.

D. Heatmap showing scaled activity scores for inferred gene modules across tumor cell Seurat clusters, with hierarchical clustering of modules and clusters. Highlighted modules correspond to programs shown in Fig. 2F.

E. Dot plot showing Hallmark pathway enrichment across inferred gene modules. Dot size indicates the number of overlapping genes, and color indicates enrichment significance.

F. UMAP feature plots showing expression of selected EGFR–ERK1/2-associated genes, including Itga2, Areg, and Ereg, across tumor cells.

bioRxiv preprint doi: https://doi.org/10.1101/2025.08.15.670583; this version posted July 24, 2026. The copyright holder for this preprint (which was not certified by peer review) is the author/funder, who has granted bioRxiv a license to display the preprint in perpetuity. It is made available under a CC-BY 4.0 International license.

Figure S3

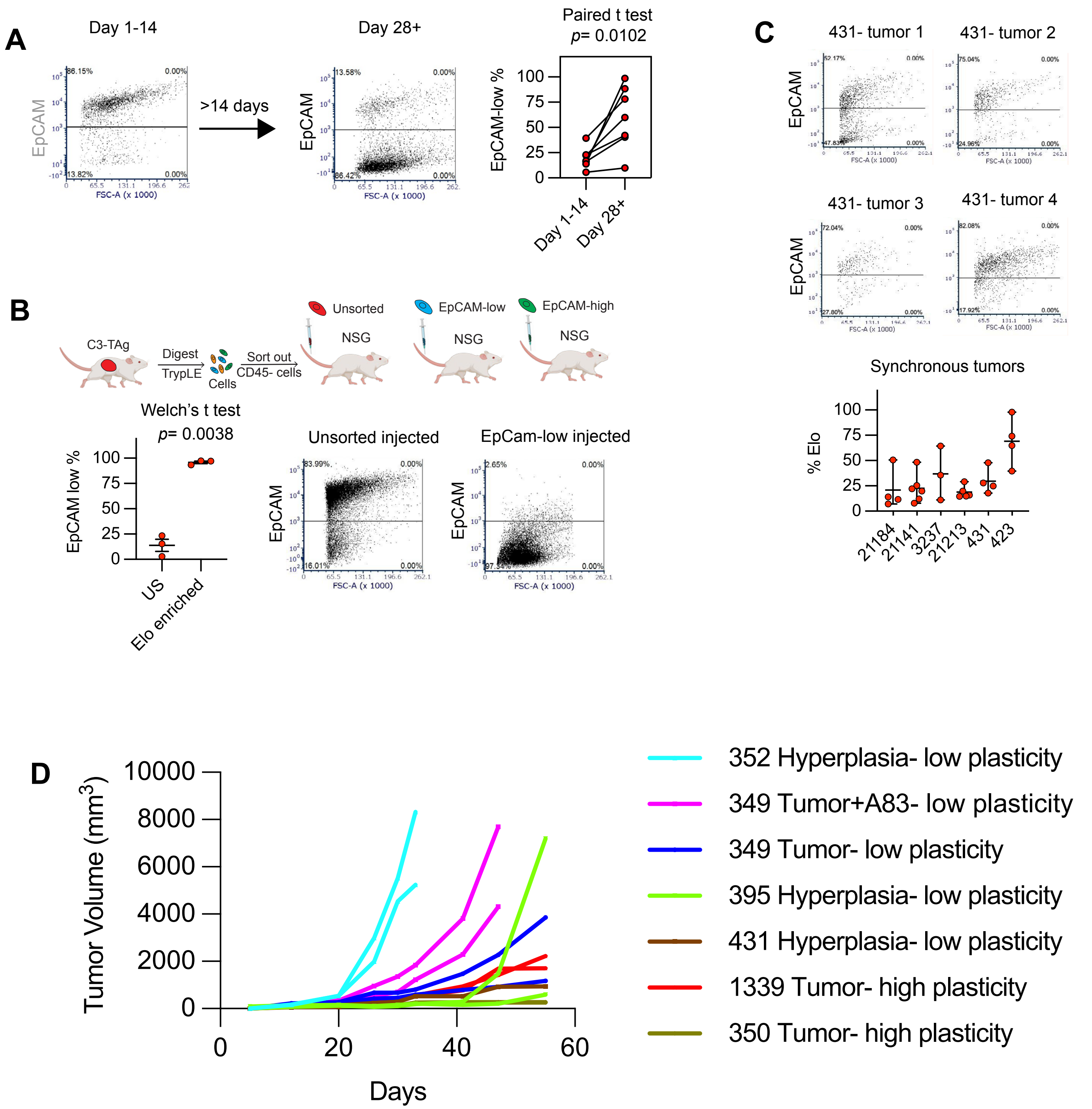

bioRxiv preprint doi: https://doi.org/10.1101/2025.08.15.670583; this version posted July 24, 2026. The copyright holder for this preprint (which was not certified by peer review) is the author/funder, who has granted bioRxiv a license to display the preprint in perpetuity. It is made available under a CC-BY 4.0 International license.

Figure S3. Related to EMT-associated plasticity is acquired during tumor progression and generates stable mesenchymal-like states.

A. Representative FACS plots and paired quantification showing the percentage of EpCAM-low cells in C3-TAg tumor organoids at early culture time points and after extended culture.

B. Experimental workflow for isolating CD45-negative unsorted, EpCAM-low, and EpCAM-high C3-TAg tumor cells and transplanting sorted populations into NSG mice. Representative FACS plots and quantification show enrichment of EpCAM-low cells in tumors derived from EpCAM-low injected cells compared with unsorted controls.

C. Representative FACS plots and quantification showing variability in the percentage of EpCAM-low cells across synchronous tumors.

D. Tumor growth curves for independent hyperplasia- and tumor-derived organoid lines classified as low- or high-plasticity based on ability to convert to an E-lo state.

bioRxiv preprint doi: https://doi.org/10.1101/2025.08.15.670583; this version posted July 24, 2026. The copyright holder for this preprint (which was not certified by peer review) is the author/funder, who has granted bioRxiv a license to display the preprint in perpetuity. It is made available under a CC-BY 4.0 International license.

Figure S4

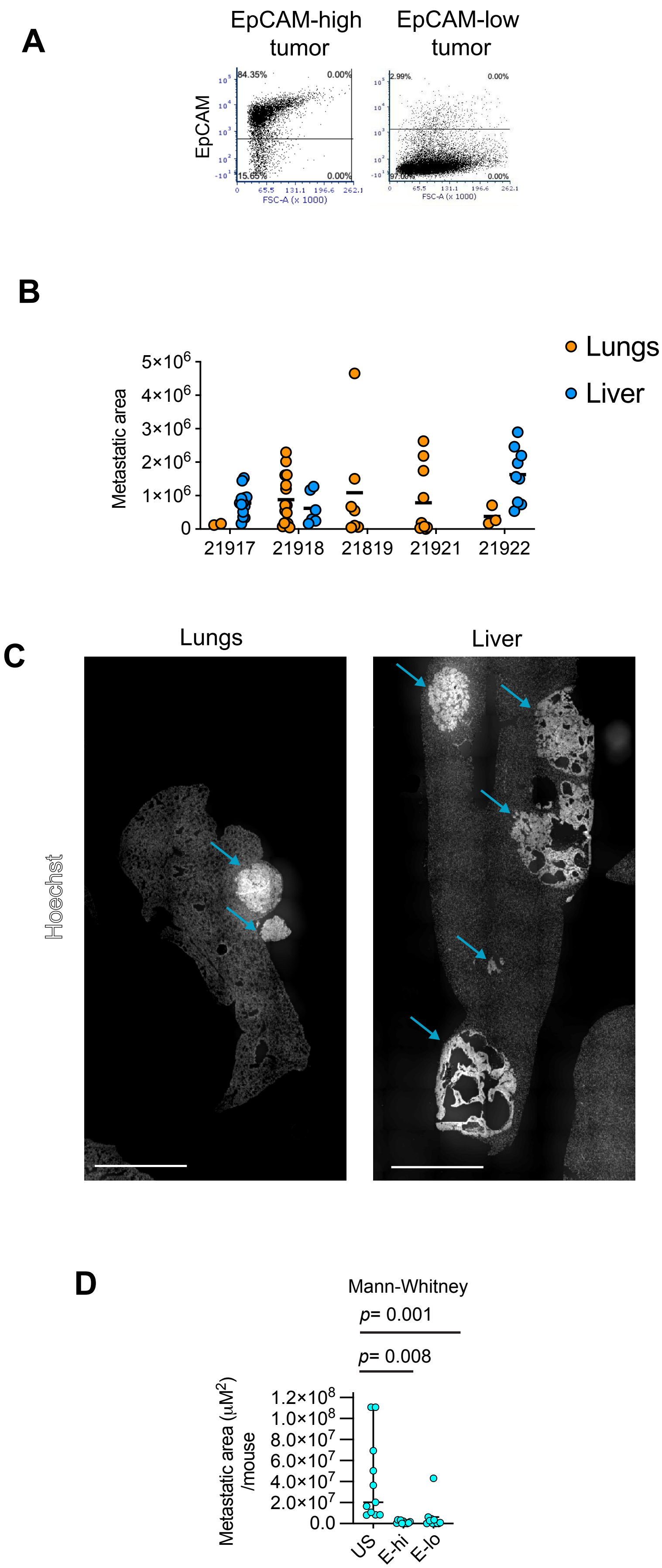

bioRxiv preprint doi: https://doi.org/10.1101/2025.08.15.670583; this version posted July 24, 2026. The copyright holder for this preprint (which was not certified by peer review) is the author/funder, who has granted bioRxiv a license to display the preprint in perpetuity. It is made available under a CC-BY 4.0 International license.

Figure S4. Related to hybrid EpCAM-high and mesenchymal-like EpCAM-low C3-TAg tumor cells are both metastatically competent.

A. Representative FACS plots showing C3-TAg tumor lines enriched for EpCAM-high or EpCAM-low tumor cell states.

B. Quantification of metastatic lesions in lungs and liver following intracardiac injection of EpCAM-high-enriched C3-TAg tumor cells. Each mouse is shown individually, with metastatic area quantified for lung and liver lesions.

C. Hoechst-stained tissue sections showing metastatic lesions in lungs and liver following intracardiac injection of EpCAM-high-enriched C3-TAg tumor cells. Arrows indicate metastatic lesions.

D. Expanded quantification from **Fig.** 4**F** that includes the Unsorted tumor cell injections. The E-hi and E-lo plots are the same data from **Fig.** 4**F**.

Quantification of metastatic area per mouse following injection of unsorted, EpCAM-high, or EpCAM-low C3-TAg tumor cells.

bioRxiv preprint doi: https://doi.org/10.1101/2025.08.15.670583; this version posted July 24, 2026. The copyright holder for this preprint (which was not certified by peer review) is the author/funder, who has granted bioRxiv a license to display the preprint in perpetuity. It is made available under a CC-BY 4.0 International license.

Figure S5

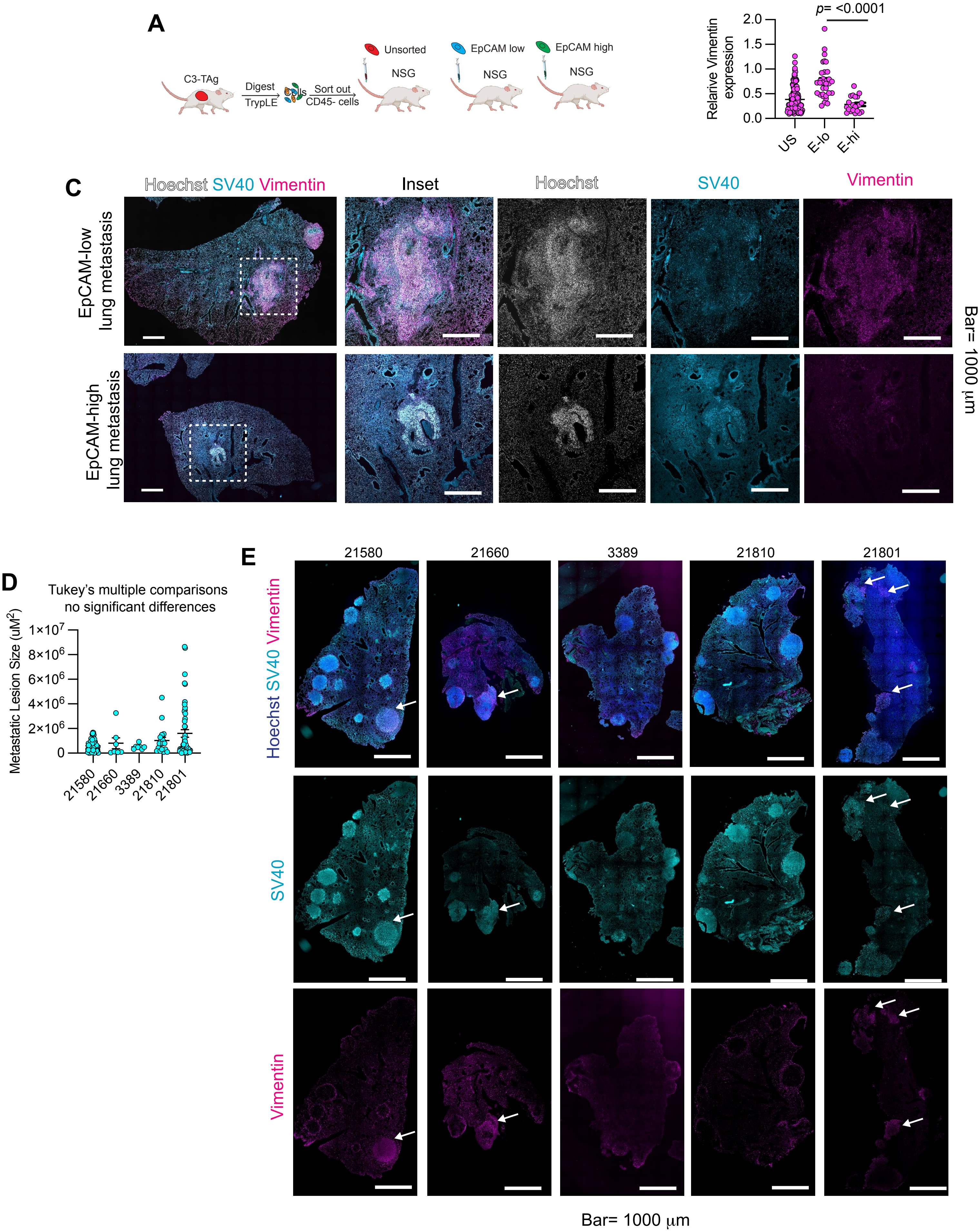

bioRxiv preprint doi: https://doi.org/10.1101/2025.08.15.670583; this version posted July 24, 2026. The copyright holder for this preprint (which was not certified by peer review) is the author/funder, who has granted bioRxiv a license to display the preprint in perpetuity. It is made available under a CC-BY 4.0 International license.

Figure S5. Related to metastatic lesions derived from EpCAM-high and EpCAM-low C3-TAg tumor cells retain distinct EMT-associated phenotypes.

A. Experimental workflow for direct FACS isolation of CD45-negative unsorted, EpCAM-high, and EpCAM-low C3-TAg tumor cells followed by injection into NSG mice and analysis of lung metastases.

B. Quantification of relative Vimentin expression in lung metastases generated by unsorted, EpCAM-low, or EpCAM-high tumor cells.

C. Representative immunofluorescence images of EpCAM-low- and EpCAM-high-derived lung metastases stained for Hoechst, SV40, and Vimentin. Scale bars, 1000 μm.

D. Quantification of metastatic lesion size across independent C3-TAg tumor lines. No significant differences were detected by one-way ANOVA with Tukey's multiple-comparisons.

E. Representative immunofluorescence images of lung metastases generated from independent C3-TAg tumor lines stained for Hoechst, SV40, and Vimentin. Arrows indicate metastatic lesions or regions of Vimentin expression. Scale bars, 1000 μm.

bioRxiv preprint doi: https://doi.org/10.1101/2025.08.15.670583; this version posted July 24, 2026. The copyright holder for this preprint (which was not certified by peer review) is the author/funder, who has granted bioRxiv a license to display the preprint in perpetuity. It is made available under a CC-BY 4.0 International license.

Table S1. Differentially expressed marker genes for tumor epithelial cell clusters. Marker genes defining each re-clustered C3-TAg tumor epithelial cell population after removal of canonical cell-cycle genes. For each gene, the table reports the associated Seurat cluster, average log2 fold change, percentage of cells expressing the gene within and outside the cluster, nominal P value, and adjusted P value.

Table S2. Hallmark pathway enrichment analysis of tumor epithelial cell clusters. MSigDB Hallmark pathway enrichment analysis performed on cluster-defining genes from re-clustered C3-TAg tumor epithelial cells. The table reports enriched Hallmark terms for each cluster, including cluster size, gene-set overlap, nominal and adjusted P values, and overlapping genes. Significant Hallmark enrichments highlight EMT-associated programs in Clusters 2, 3, 6, and 8.

Table S3. Gene Ontology enrichment analysis of tumor epithelial cell clusters. Gene Ontology enrichment analysis of cluster-defining genes from re-clustered C3-TAg tumor epithelial cells. The table lists enriched GO biological process terms for each cluster, including cluster size, gene-set overlap, nominal and adjusted P values, and overlapping genes. These enrichments support functional differences among tumor epithelial states, including programs associated with proliferation, epithelial differentiation, EMT, motility, and extracellular matrix organization.

Table S4. Gene module assignments for tumor epithelial cells. Gene module assignments derived from tumor epithelial cell trajectory analysis. The table lists each gene, its assigned module and supermodule, and the corresponding dimensional coordinates used to organize module structure.

Table S5. Hallmark pathway enrichment analysis of tumor epithelial gene modules. MSigDB Hallmark pathway enrichment analysis performed on genes assigned to each tumor epithelial cell module. The table reports enriched Hallmark terms for each module, including module size, gene-set overlap, nominal and adjusted P values, and overlapping genes.

Table S6. Gene Ontology biological process enrichment analysis of tumor epithelial gene modules. Gene Ontology biological process enrichment analysis performed on genes assigned to each tumor epithelial cell module. The table lists enriched biological process terms for each module, including module size, overlap, nominal and adjusted P values, and overlapping genes.

Table S7. Tabula Muris cell-type enrichment analysis of tumor epithelial gene modules. Enrichment analysis comparing tumor epithelial gene modules with Tabula Muris cell-type signatures. The table reports enriched cell-type terms for each module, including module size, gene-set overlap, nominal and adjusted P values, and overlapping genes.

Table S8. Gene Ontology cellular component enrichment analysis of tumor epithelial gene modules. Gene Ontology cellular component enrichment analysis performed on tumor epithelial gene modules. The table lists enriched cellular component terms for each module, including module size, overlap, nominal and adjusted P values, and overlapping genes.

bioRxiv preprint doi: https://doi.org/10.1101/2025.08.15.670583; this version posted July 24, 2026. The copyright holder for this preprint (which was not certified by peer review) is the author/funder, who has granted bioRxiv a license to display the preprint in perpetuity. It is made available under a CC-BY 4.0 International license.

Table S9. Gene Ontology molecular function enrichment analysis of tumor epithelial gene modules. Gene Ontology molecular function enrichment analysis performed on tumor epithelial gene modules. The table reports enriched molecular function terms for each module, including module size, overlap, nominal and adjusted P values, and overlapping genes.

Table S10. Enrichment of tumor epithelial gene modules for TGFβ- and EGFR–ERK1/2-regulated programs. Enrichment analysis comparing tumor epithelial gene modules with gene signatures induced by EGF or TGFβ treatment and genes suppressed by erlotinib or trametinib in C3-TAg tumor organoids. The table includes enrichment scores, nominal and FDR-adjusted P values, overlap counts, and overlapping genes.

Table S11. Enrichment of tumor epithelial clusters for TGFβ- and EGFR–ERK1/2-regulated programs. Enrichment analysis comparing tumor epithelial Seurat cluster gene signatures with TGFβ-induced, EGF-induced, erlotinib-suppressed, and trametinib-suppressed gene sets from C3-TAg tumor organoids. The table reports overlap counts, nominal P values, and enrichment significance for each cluster-signature comparison.
